# Retraction: BMSC-derived exosomes carrying microRNA-122-5p promote progression of osteoblasts in osteonecrosis of the femoral head

**DOI:** 10.1042/CS-2018-1064_RET

**Published:** 2024-10-16

**Keywords:** bone marrow mesenchymal stem cell, exosome, microRNA-122-5p, osteonecrosis of the femoral head, RTK/Ras/MAPK signaling pathway, sprouty2

This article “BMSC-derived exosomes carrying microRNA-122-5p promote proliferation of osteoblasts in osteonecrosis of the femoral head” DOI: 10.1042/CS20181064 is being retracted from *Clinical Science* at the request of the Editor-in-Chief and the Editorial Board following receipt of a notification from a reader, alerting the Editorial Board to concerns surrounding the scientific validity of the flow cytometry histograms in Figure 8E and Figure 9A. The Editorial Office also identified undeclared replacements of figure images during the revision process of this article. The authors have been contacted with regards to the retraction and have not responded to the Journal's queries or the concerns raised. The corresponding author's institution Wuhan Third Hospital was contacted. The institution responded, but the information provided did not alleviate the concerns raised. Additionally, the institution stated that the flow cytometry experiments were conducted by a third-party company, which was not declared in the article or during the submission process. Given the extent of the issues raised, the Editorial Board stand by the decision to retract the article.

